# *SARS‐CoV‐2* (COVID‐19) pandemic and a possible impact in the future of menstrual cycle research

**DOI:** 10.1002/hsr2.276

**Published:** 2021-05-03

**Authors:** Raul Cosme Ramos Prado, Rodrigo Silveira, Ricardo Yukio Asano

**Affiliations:** ^1^ Women's Science Studies and Research Academy São Paulo Brazil; ^2^ Exercise Psychophysiology Research Group, School of Arts, Sciences and Humanities University of Sao Paulo Sao Paulo Brazil; ^3^ School of Physical Education and Sport University of Sao Paulo Sao Paulo Brazil

Placental mammals (eg, primates, canines, rodents) are species known to have a reproductive cycle of diverse length. The length of a dog's estrous cycle range from 140 to 350 days,^1^ whilst for mice and rats the mean is of 4 days,^2^ another example is a range between 28 and 32 days in *Rhesus* monkeys' cycle.^3^ In female *Homo sapiens*, robust data from a recent study with 124 648 women, from which 612 613 showed regular cycles of 29.3 ± 5.2 days.^4^

The female human menstrual cycle (MC) can be classified into general (ie, follicular and luteal) or sub‐phases (eg, menses, early‐to‐late follicular, ovulatory, mid‐to‐late‐luteal) based on sexual hormones fluctuation (ie, progesterone and estrogen). MC is orchestrated by the hypothalamic‐pituitary‐ovarian (HPO) axis through a positive and negative feedback loop of hormones on these structures.^5^ The first day of the cycle is visibly characterized by menstruation (ie, bleeding of superficial endometrium). During the early days of the cycle, there is a slight increase of pituitary secretes (follicle‐stimulating and luteinizing hormone) acting on ovaries to stimulate the growth of follicles. At a similar pace, the follicle secret estrogen and progesterone to stimulate adaptations in the uterus and breasts for fertilization. At the ovulatory period, the high‐surge of estrogen and the slight increase of progesterone stimulate the high secretion of LH (surge) by pituitary glands (positive feedback) releasing now mature ovum. After ovum liberation (mid‐to‐late‐luteal), the follicle is converted in corpus luteum increasing progesterone secretion and basal body temperature (~0.5°C ‐ thermogenic effect of progesterone) at the point that if fertilization does not occur progesterone levels fall and a new cycle begins.^5^

The MC is a biological event that fills a large share of scientific studies, including animals^1^, ^2^, ^3^ and humans.^4^, ^6^, ^7^ Human's MC investigators describe that monitoring/identifying specific MC phases are among the oldest challenges in this field.^8^, ^9^ Methods as: retrospective or prospective self‐report; hormone dosage; basal body temperature control and other strategies compete between accuracy vs cost to identify the MC phase. For example, self‐report methods have a lower cost but they offer worse accuracy (mainly in the middle‐end of the cycle), in contrast, sonography has an excellent accuracy but is accompanied by the high cost.^9^

Independent of the method used to investigate the MC, it is clear (with exceptions) that eumenorrheic (regular) MC is necessary to control the study, in which variations in its length can reduce the identification accuracy of a specific phase of the cycle. As a consequence, the internal success of the research can be impaired. Therefore, researchers need to be aware of any unforeseen changes in women's life that could make the MC unpredictable, besides controlling their experimental designs.

A new year came but, with it, we are experiencing one of the greatest pandemics of history, with the sad mark of approximately 117 million infected people and more than 2.6 million deaths caused by complications of *SARS‐CoV‐2* (COVID‐19) until March 11, 2021.^10^ With these alarming numbers, several studies^11^, ^12^, ^13^ have been published showing changes in people's behavior during the COVID‐19 pandemic. Specifically, women have reported significantly higher stress, anxiety, and depression compared to men.^11^, ^13^ A cohort (1994 to 2014) and multicentric (30 countries) study^14^ showed that negative psychological responses are prevalent in women. These findings, combined with COVID‐19 studies,^11^, ^13^ reinforce the idea that negative psychological changes have been exacerbated during the pandemic, mainly for women.

It has been supported that extrinsic factors can cause irregularities in the length of MC. According to previous studies, HPO is sensible to stressors (eg, inadequate sleep, level of psychological and physical stress) causing changes in the pulsating mechanisms of hormones and in the duration of MC.^15^, ^16^, ^17^ Therefore, it is plausive to accept that the fear of what we are living and the uncertainty about where are we going after the current pandemic has made our lives more unpredictable and have been changing the regularity of our system.

Early results in the exercise science field showed that one among five female athletes experienced changes in MC length during the COVID‐19 pandemic.^18^ There is a hypothesis that these amenorrheic cycles can be consequences of the psychological stress impact of the COVID‐19 in HPO.

From a scientific viewpoint, the current pandemic generates a promising way to investigate human behavior, technologies, vaccines, and methods against COVID‐19. In contrast, it is a disaster for the controlled MC research, once there is a concern in this field that needs female cycles to be predictable.

The effect of the COVID‐19 pandemic on MC is still unclear in the general women population, but there is a chance that early results found in female athletes^18^ occur also in other groups of women worldwide, weakening the research methodological control and taking twice as long to present the outcomes. It would reduce the number of evidences and cause resources waste. This consideration is threatening for the future of MC science once in the current pandemic the mask of some country's leaders has fallen showing the face of scientific negationism, which created cuts and barriers to research investment.^19^ Therefore, we suggest that future studies seek to confirm the above hypothesis of irregularities on MC length.

Finally, as MC investigators, we reinforce that researchers need to be aware and prepared (one more time) to understand that in the near‐future women may suffer a COVID‐19 pandemic “*post‐traumatic*” period that will make the irregularity of the MC common between them. These facts will force methodological adaptations for the control of the MC, otherwise, years of financial investment in research and their contributions to women's science will be compromised.

## CONFLICT OF INTEREST

The authors have no conflict of interest.

## AUTHOR CONTRIBUTIONS

Writing – conception and original draft: Raul Prado

Writing – review and editing: Raul Prado, Rodrigo Silveira and Ricardo Asano.

## TRANSPARENCY STATEMENT

Raul Cosme Ramos do Prado affirms that this manuscript is an honest, accurate, and transparent account of the study being reported; that no important aspects of the study have been omitted; and that any discrepancies from the study as planned (and, if relevant, registered) have been explained.

## Data Availability

Data sharing is not applicable to this article as no new data were created or analyzed in this study.
